# Supplementary material for: Understanding Physicochemical Mechanisms of Sequential Infiltration Synthesis toward Rational Process Design for Uniform Incorporation of Metal Oxides
Source: Sensors (Basel). 2022 Aug 16;22(16):6132. doi: 10.3390/s22166132 (PMC9416371; doi:10.3390/s22166132)
Supplement: Supplementary file 1 [file sensors-22-06132-s001.zip › sensors-1809134-supplementary.pdf]

Supporting Information:

Understanding Physicochemical Mechanisms of Sequential Infiltration Synthesis toward Rational Process Design for Uniform Incorporation of Metal Oxides

Jiwoong Ham,[1] *Minkyung Ko*,[1] *Boyun Choi*,[1] *Hyeong-U Kim*,[2] and *Nari Jeon*[1,*]

[1] Department of Materials Science and Engineering, Chungnam National University, Daejeon 34134, Republic of Korea

[2] Department of Plasma Engineering, Korea Institute of Machinery & Materials (KIMM), Daejeon 34103, Republic of Korea

Corresponding author: njeon@cnu.ac.kr

1. Experimental Conditions

Table S1. Precursor dose, exposure, and purge time for the data used in Figure 1.

Figure 1 Temperature [°C]	TMA			H_2O		
	Dose [s]	Exposure [s]	Purge [s]	Dose [s]	Exposure [s]	Purge [s]
80	2	1800	1200	2	600	600

Table S2. Precursor dose, exposure, and purge time for the data used in Figure 2.

Figure 2	TMA		
Temperature [°C]	Dose [s]	Exposure [s]	Purge [s]
80	2	2450	5
90	2	610	5
100	2	260	5
110	2	52	5
120	2	26	5
130	2	3	5
140	2	300	5
155	2	300	5
170	2	300	5
185	2	300	5
200	2	300	5

Table S3. Precursor dose, exposure, and purge time for the data used in Figure 3.

Figure 3a	TMA		
Temperature [°C]	Dose [s]	Exposure [s]	Purge [s]
80	2	4000	5
90	2	4000	5
100	2	3500	5
110	2	2000	5
120	2	1500	5
130	2	600	5

Figure 3b		TMA	
Temperature [°C]	Dose [s]	Exposure [s]	Purge [s]
80	2	1800	10000
90	2	610	6000
100	2	260	2500
110	2	52	2000
120	2	26	1500
130	2	3	1000

Table S4. Precursor dose, exposure, and purge time for the data used in Figure 4.

Figure 4a		TMA			H_2O	
Temperature [°C]	Dose [s]	Exposure [s]	Purge [s]	Dose [s]	Exposure [s]	Purge [s]
80	2	2450	5	2	600	600
90	2	610	5	2	600	600
100	2	260	5	2	600	600
110	2	52	5	2	600	600
120	2	26	5	2	600	600
130	2	3	5	2	600	600
**Figure 4b**		**TMA**			**H_2O**	
**Temperature [°C]**	**Dose [s]**	**Exposure [s]**	**Purge [s]**	**Dose [s]**	**Exposure [s]**	**Purge [s]**
80	2	2450	1516	2	600	600
90	2	610	636	2	600	600
100	2	260	248	2	600	600
110	2	52	160	2	600	600
120	2	26	97	2	600	600
130	2	3	65	2	600	600

Table S5. Precursor dose, exposure, and purge time for the data used in Figure 5.

Single Temp. 1cy		TMA			H_2O		Number of
Temperature [°C]	Dose [s]	Exposure [s]	Purge [s]	Dose [s]	Exposure [s]	Purge [s]	cycles
80	2	1800	600	2	600	600	1
**Single Temp. 5cy**		**TMA**			**H_2O**		**Number of cycles**
**Temperature [°C]**	**Dose [s]**	**Exposure [s]**	**Purge [s]**	**Dose [s]**	**Exposure [s]**	**Purge [s]**	
80	2	1800	600	2	600	600	5
**Two Temp. 5cy**		**TMA**			**H_2O**		**Number of cycles**
**Temperature [°C]**	**Dose [s]**	**Exposure [s]**	**Purge [s]**	**Dose [s]**	**Exposure [s]**	**Purge [s]**	
80	2	1800	600	2	600	600	1
130			2500 (Heating and stabilization)				1
130	2	600	600	2	600	600	4

Table S6. Precursor dose, exposure, and purge time for the data used in Figure 6.

Single Temp. 5cy	TMA			H_2O			Number of cycles
Temperature [°C]	Dose [s]	Exposure [s]	Purge [s]	Dose [s]	Exposure [s]	Purge [s]	
80	2	1800	600	2	600	600	5

Two Temp. 5cy	TMA			H_2O			Number of cycles
Temperature [°C]	Dose [s]	Exposure [s]	Purge [s]	Dose [s]	Exposure [s]	Purge [s]	
80	2	1800	600	2	600	600	1
130	2500 (Heating and stabilization)						1
130	2	600	600	2	600	600	4

2. *in* situ FTIR Spectroscopy

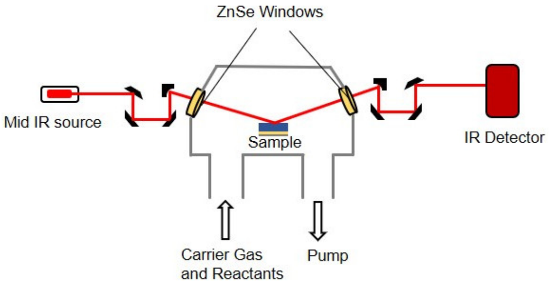

Figure S1. Schematic of the *in situ* FTIR spectrometer setup connected with a thermal ALD reactor.

3. Analysis of diffusion constant

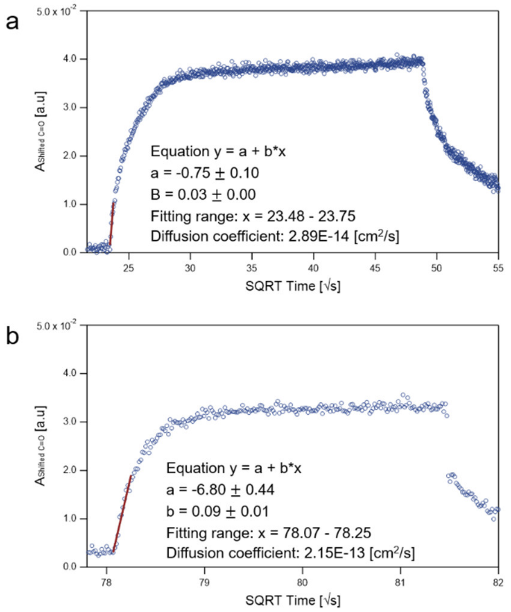

Figure S2. Calculation of diffusion coefficients in the 1st TMA exposure step when growth temperatures are (a) 80 °C and (b) 100 °C

4. XPS analysis at different etch levels

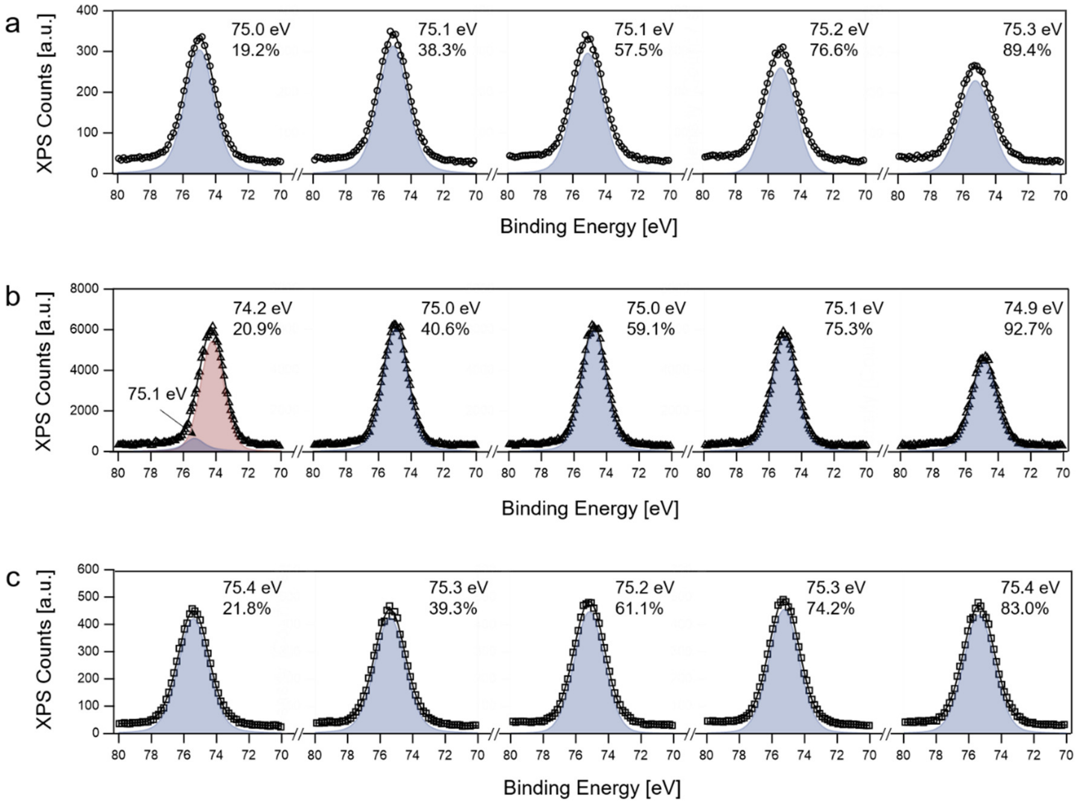

Figure S3. HRXPS at different etch levels for (a) single-temperature one-cycle SIS, (b) single-temperature five-cycle SIS, and (c) two-temperature five-cycle SIS. Each etch level (see **Figure 4(a)**) is denoted in the corresponding panels

5. Effects of traces of water

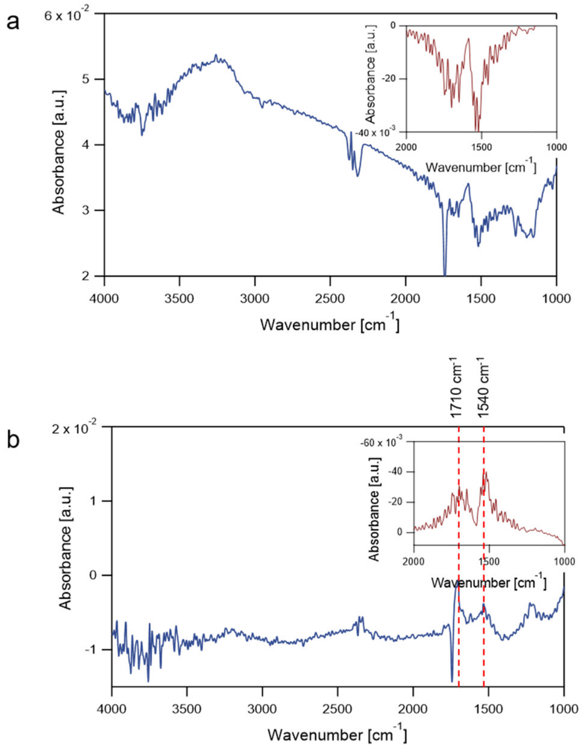

Figure S4. Absorbance spectra collected at the end of the 5th H_2O purge step (a) in two-temperature five-cycle SIS experiments and (b) in the single-temperature five-cycle SIS. Both spectra were compared with the absorbance spectrum (inset) from a pristine PMMA collected after the temperature was increased from 80 °C to 130 °C (background spectrum: a pristine PMMA at 80 °C). The inset of (b) is drawn with an inverted Y-axis.

The inset of Figure S4(a) displays the loss of the H_2O molecules (i.e., negative peaks) in the reaction chamber. Comparing the spectra in **Figure S4(a)** and its **inset** indicates that the peaks in the spectra in the range of 2000 – 1000 cm^{-1} in **Figure S4(a)** are due to the decreased concentration of H_2O molecules in the chamber. The absorbance spectrum shown in **Figure S4(b)** is similar to that given in the **inset**, which is drawn with an inverted Y-axis for a better comparison with it in the same wavenumber range (2000 – 1000 cm^{-1}) in **Figure S4(b)**. The peaks in the range of 2000 – 1000 cm^{-1} in **Figure S4(b)** originate from the H_2O molecules present at an increased concentration in the reaction chamber.

6. Single-temperature five-cycle SIS at 80 °C

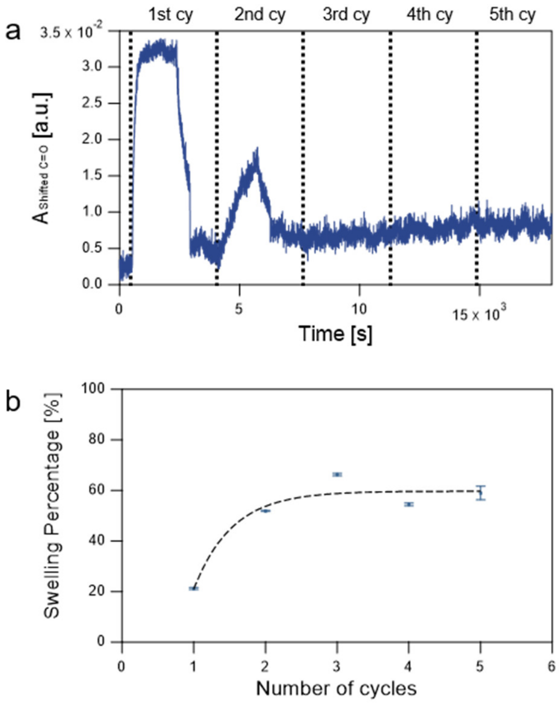

Figure S5. (a) Absolute values of the absorbance of the shifted C=O band as a function of time and (b) swelling percentage as a function of the number of SIS cycles in the single-temperature SIS experiment at 80 °C

7. Background fitting process

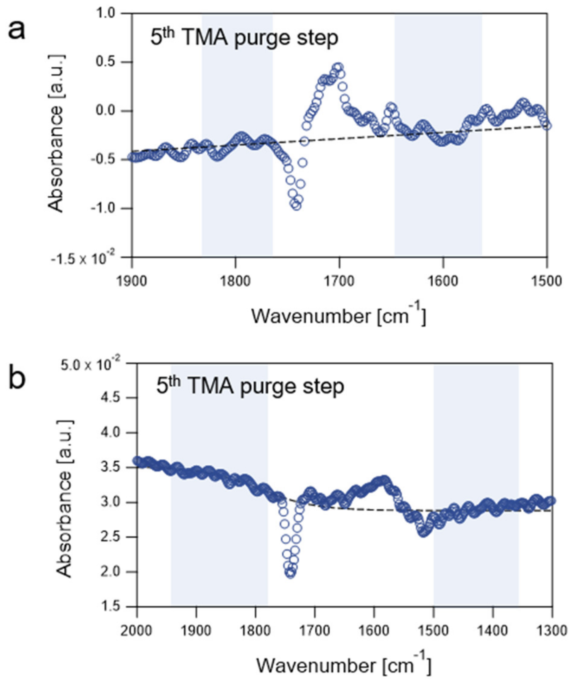

Figure S6. Examples of background fitting processes for (a) single-temperature five-cycle SIS and (b) two-temperature five-cycle SIS experiments. The wavenumber range used to fit the baseline is represented by blue-shaded areas. The fitted background lines are shown as dashed lines.

The aforementioned examples show the absorbance spectra collected at the end of the 5th TMA purge steps in each SIS experiment. A linear function was used to fit the background of the absorbance spectra from the single-temperature five-cycle SIS experiment (**Figure S6(a)**). A sigmoid function was used to fit the background of the absorbance spectra from the two-temperature five-cycle SIS experiment (**Figure S6(b)**).

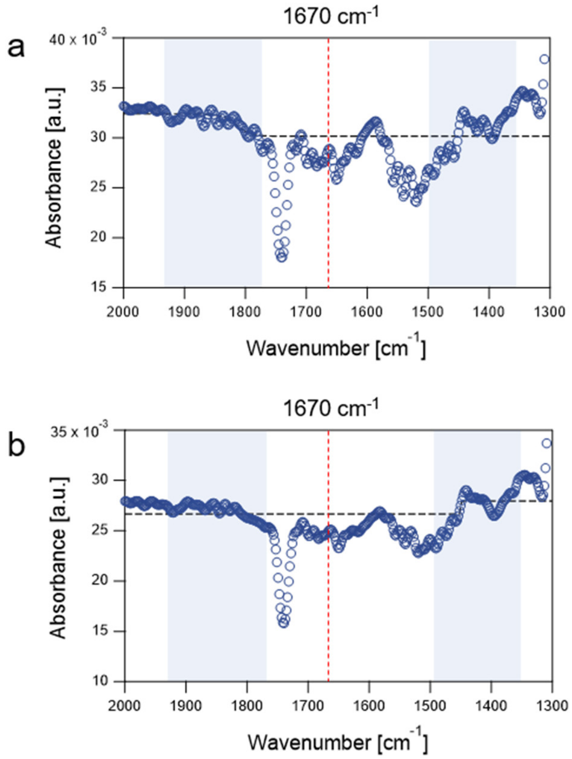

Figure S7. Absorbance spectrum recorded at the end of (a) the 4th and (b) the 5th TMA exposure steps in the two-temperature five-cycle SIS experiment. The wavenumber range used to fit the baseline is represented by blue-shaded areas. The fitted background lines are shown as dashed lines.

As shown in **Figure S7**, the absolute values of the absorbance of the shifted C=O band (1670 cm^{-1}) in the 4th and the 5th TMA exposure steps were nil, similar to the noise level.
